# Gamma Knife Radiosurgery Followed by Flow-Reductive Embolization for Ruptured Arteriovenous Malformation

**DOI:** 10.3390/jcm9051318

**Published:** 2020-05-02

**Authors:** Myung Ji Kim, So Hee Park, Keun Young Park, Hyun Ho Jung, Jong Hee Chang, Jin Woo Chang, Jae Whan Lee, Won Seok Chang

**Affiliations:** 1 Departmenf of Neurosurgery, Yonsei University College of Medicine, Seoul 03722, Korea; KMJ8686@yuhs.ac (M.J.K.); SHPARK123@yuhs.ac (S.H.P.); KYPARK78.MD@yuhs.ac (K.Y.P.); JUNGHH@yuhs.ac (H.H.J.); CHANGJH@yuhs.ac (J.H.C.); JCHANG@yuhs.ac (J.W.C.); 2 Brain Research Institute, Yonsei University College of Medicine, Seoul 03722, Korea

**Keywords:** arteriovenous malformation (AVM), cerebrovascular disease (CVD), embolization, gamma knife radiosurgery (GKRS), intracerebral hemorrhage, radiosurgery, Spetzler-Martin grade

## Abstract

Background: Aggressive treatment to achieve complete obliteration of brain arteriovenous malformation (AVM) is necessary in patients with a recent history of hemorrhage. The major drawback of Gamma knife radiosurgery (GKRS) alone for AVM is risk of bleeding during the latent period until the AVM occludes. At our center, patients who present with ruptured AVMs are frequently offered GKRS followed by embolization. The goal of this study was to compare outcomes of embolization for patients who have previously undergone GKRS for ruptured AVMs. Methods: A database including 150 GKRS for ruptured AVMs between November 2008 and October 2017 was reviewed. The embolized group was selected by including AVMs with post-GKRS embolization. The non-embolized group was defined as AVMs treated by GKRS alone. Outcomes including obliteration rate, incidence of repeat hemorrhage, and delayed cyst formation were compared between two groups. The predictive factors related to AVM obliteration and complications were analyzed. Results: The study consisted of 81 patients in the non-embolized group and 17 patients in the embolized group. Statistically significant differences were detected between the two groups with respect to age, Pollock-Flickinger score, Spetzler-Martin (SM) grade, eloquence of adjacent brain, and presence of aneurysms. The embolized group included more AVMs with larger median nidus volume. The predictive factors for the obliteration of ruptured AVMs were nidus volume, SM grade, Virginia Radiosurgery AVM Scale (VRAS), and Pollock-Flickinger score and for the subsequent hemorrhage were marginal dose, nidus volume, SM grade, VRAS, and Pollock-Flickinger score. The obliteration rates and complication rates after GKRS between groups were not significantly different. However, this study demonstrated statistically significant difference in the cumulative incidence of obliteration in AVMs with SM grade III and IV (*p* = 0.037). Conclusion: Although the current study demonstrated similar results in patients who underwent GKRS with and without embolization, the embolized group included more AVMs with larger nidus volume, higher SM grade, Pollock-Flickinger score, and aneurysm, which have a lower chance of obliteration and a higher probability of repeat hemorrhage. GKRS followed by embolization appears to be a beneficial approach for the treatment of ruptured AVMs that are at risk for obliteration failure and repeat hemorrhage during the latency period after single-session GKRS alone. Further studies involving a larger number of cases and continuous follow-up are necessary to confirm our conclusions.

## 1. Introduction

Intracranial hemorrhage is the most devastating complication of intracranial arteriovenous malformations (AVM). Without treatment, the overall risk of a spontaneous hemorrhage from a brain AVM has been reported to range from 2% to 5% per year [1,2,3]. The risk may increase with intranidal aneurysms, deep venous drainage, a single draining vein, venous stenosis, high mean arterial pressure in the feeder, diffuse morphology, periventricular location, and larger nidus size [4,5,6]. However, morbidity and mortality rates associated with hemorrhage are very high [7]. Furthermore, initial AVM rupture has been shown to increase the risk of subsequent rupture [4]. Previous hemorrhage has been reported as one of the significant factors for further bleeding [1,8] and aggressive treatment to achieve complete obliteration is necessary in patients with a recent history of hemorrhage [9]. The treatment options include endovascular embolization, gamma knife radiosurgery (GKRS), surgical resection, and multimodality approaches using these three techniques. GKRS has been established as an effective treatment option for intracranial AVMs, especially with small or medium nidi located in eloquent or deep regions [10,11,12]. The major drawback of radiosurgical AVM treatment is the risk of bleeding during the latent period until the AVM is occluded [13]. This limitation is most critical for ruptured AVMs, which have a high subsequent rupture risk within the first year after hemorrhage. GKRS and embolization are increasingly being used in combination for larger, complicated AVMs and especially for ruptured AVMs. The benefits of embolization before GKRS include reduced AVM volume, allowing application of higher radiation dose to the margin of the smaller target volume with a better obliteration rate and fewer complications [10]; eradicating angiographic predictors of hemorrhage [14]; and reducing symptoms associated with arterial steal or venous hypertension [15]. However, several studies have demonstrated that pre-GKRS embolization may be disadvantageous in treating AVMs. Pre-GKRS embolization may obscure brain AVM nidus delineation, both by superimposition of embolic material [1] and the presence of collateral feeding vessels [16]. A shielding effect caused by embolization materials with high atomic mass has also been suggested to obscure brain AVM nidus delineation [17]. Lastly, embolization may induce hypoxia, making the AVM tissue less radiosensitive and increasing its angiogenic activity [18].

At our facility, patients who present with an intracranial hemorrhage requiring hematoma evacuation and decompression are candidates for surgical resection. Patients who have been ruled out as surgical candidates are offered GKRS followed by embolization, instead of GKRS after embolization due to the several disadvantages described above. After a short interval, following endovascular embolization can be used to treat the remaining AVM. During the post-GKRS period, the risk of AVM repeat rupture has been reduced by endovascular embolization. In this study, we report the results of a retrospective analysis of outcomes for all patients who presented to our institution with ruptured AVMs. All included patients were treated with GKRS before endovascular embolization or with GKRS alone. 

## 2. Methods

### 2.1. Patient Characteristics

A database including 150 cases of patients with ruptured AVMs who underwent GKRS between November 2008 and October 2017 at our hospital was retrospectively reviewed. The embolized group comprised of AVMs with post-GKRS embolization. The non-embolized group was defined as AVMs treated by GKRS alone. All patients presented with hemorrhage confirmed by non-contrast enhanced computed tomography (CT) imaging. This study excluded patients who had follow-up for less than 2 years or who had been previously treated by resection or embolization in our hospital or at another institution. All patients had at least one post-GKRS magnetic resonance imaging (MRI) or cerebral angiography follow-up study at our hospital available for analysis. Patient clinical data, including demographic characteristics, imaging findings before embolization and GKRS, radiosurgical parameters, and follow-up images, were reviewed. Spetzler-Martin (SM) grade, Virginia Radiosurgery AVM Scale (VRAS), and Pollock-Flickinger score were used to classify AVMs. This study obtained full ethical approval from the institutional review board. 

### 2.2. Gamma Knife Radiosurgery 

The procedure for GKRS has been previously described [19,20]. In brief, all patients underwent stereotactic frame placement and stereotactic planning neuroimaging, using MRI and digital subtraction angiography (DSA). Radiosurgery was performed using the Leksell Gamma Knife Perfexion Unit (Elekta AB, Elekta Company, Stockholm, Sweden). The AVM nidus was delineated using stereotactic MRI, including T1-weighted contrast-enhanced and T2-weighted imaging sequences, and DSA. Dose planning was performed by a neurosurgeon and a medical physicist depending on AVM nidus location and calculated lesion volume. 

### 2.3. Endovascular Embolization

Planning of endovascular treatment was based on consensus by a multidisciplinary stroke team meeting after the careful evaluation of 3-dimentional angiograms. Endovascular embolization was performed for the purpose of reducing nidus flow and decreasing the risk of subsequent hemorrhage during the latent period. The embolized group underwent transarterial embolization using N-butyl cyanoacrylate (NBCA) or non-adhesive copolymer ethylene vinyl alcohol (Onyx; Medtronic, Irvine, California, CA, USA) at our institution. Post-GKRS embolization was scheduled for the earliest possible date after GKRS, considering the patients’ medical and neurologic conditions. 

### 2.4. Neuroimaging Follow-up and Outcomes Assessment

After GKRS, all patients underwent clinical evaluation and MRI/MR angiography (MRA) including time-of-flight (TOF) studies at 6-month intervals for the first 2 years and then annually thereafter. Once the nidus was considered obliterated on MRI/MRA, a cerebral angiogram was recommended. Total obliteration of the AVM was defined as complete absence of nidus filling on angiogram [20]. If the patient missed an angiogram, the AVM was considered obliterated if there was no flow void on MRI or vascular filling on MRA [20]. If obliteration of the AVM nidus was not observed within 3–4 years after initial GKRS, salvage treatments for residual nidi were recommended. The AVM nidus was measured at treatment and after treatment using the latest available follow-up MRI scan [9]. For this group of patients who underwent repeat GKRS, images from the initial treatment and images before the repeat GKRS were used for analysis. Measurements were made on T1-weighted contrast-enhanced imaging. The nidus was defined as the contrast-enhancing portion of the AVM, excluding any draining veins or arterial feeders [9]. Delayed cyst formation was defined as a fluid-filled cavity with an enhancing nodular lesion on T1-weighted contrast-enhanced imaging following GKRS for AVM. Incidence of repeat hemorrhage and delayed cyst formation were assessed using MRI throughout follow-ups. In case of presentation of any neurologic symptoms, an emergent non-contrast enhanced CT imaging was performed to reveal any complications. 

### 2.5. Statistical Analysis 

The Chi-square test, Fisher exact test, and Mann-Whitney U-test were used to examine differences between the embolized and non-embolized groups. Descriptive statistics are presented as the median and range for continuous variables and as frequency and percentage for categorical variables. Since all continuous variables did not satisfy normality, they were analyzed by the Mann-Whitney U-test. Descriptive statistics were presented as medians (Q1: cumulative percentage of 25%, Q3: cumulative percentage of 75%). The rates of obliteration, repeat hemorrhage, and delayed cyst formation after treatment were assessed in both groups. Obliteration and complication rates were compared between the two groups using Chi-square test or Fisher’s exact test, as appropriate. The Kaplan-Meier method was used to investigate different groups in association with the obliteration and complication rates after stratification with the log-rank test. Univariate analyses were performed using the Cox proportional hazards regression model to analyze the predictive factors of AVM obliteration and complications. The hazard ratio (HR) and 95% confidence interval (CI) were calculated. Statistical significance was indicated by a *p* value less than 0.05. All analyses were performed using statistical software (SAS, version 9.4, SAS Inc., Cary, NC, USA and R package, version 3.6.0).

## 3. Results

### 3.1. Patient Demographics, AVM Characteristics, and Radiosurgical Parameters

Among 150 GKRS for ruptured AVMs, 14 cases of repetitive GKRS were identified, therefore a total of 136 patients underwent GKRS for ruptured AVMs. Fourteen patients were excluded because their follow-up periods were less than 2 years and they did not undergo follow-up imaging studies. Twenty-four patients who underwent embolization or surgical resection before GKRS were also excluded. In total, 98 patients were enrolled in this study. The non-embolized group consisted of 81 patients and the embolized group consisted of 17 patients.

The median interval between embolization and GKRS was 6.3 days (range 0–21 days). The embolic agents were Onyx, used in 12/17 procedures (70.6%), and NBCA, used in 5/17 procedures (29.4%). Table 1 presents patient demographics, AVM characteristics, and radiosurgical parameters in detail. Statistically significant differences were detected between the two groups with respect to age, Pollock-Flickinger score, SM grade distribution, eloquence of adjacent brain, and presence of aneurysms. None of the patients in the non-embolized group had AVMs with flow-related aneurysm. The embolized group included more AVMs with larger median nidus volume, although it was not significantly different between the two groups.

### 3.2. Imaging Outcome—AVM Obliteration

**Figure 1:**
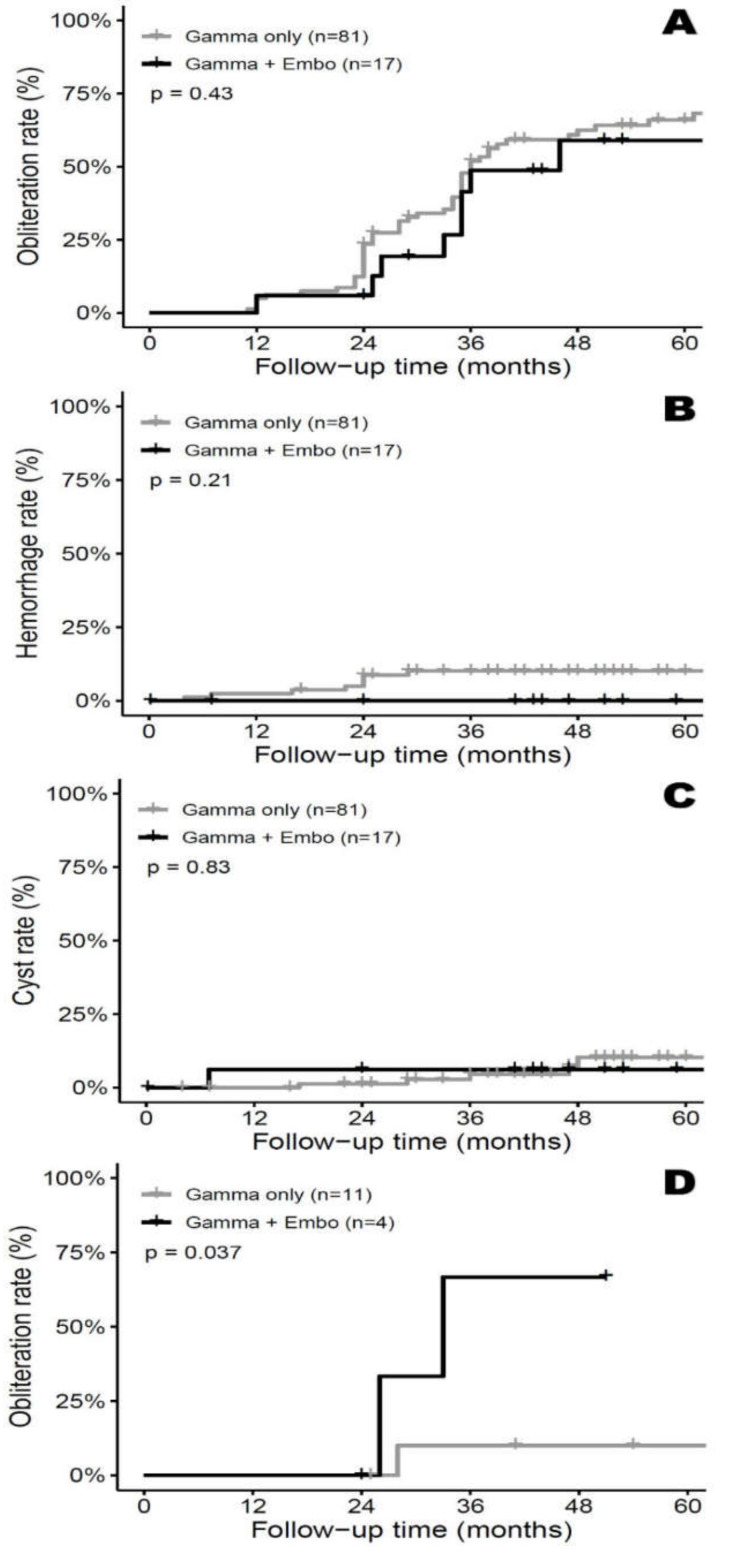
(**A**) Kaplan-Meier curves for total obliteration confirmed by magnetic resonance (MR) imaging/MR angiography or cerebral angiogram after gamma knife radiosurgery (GKRS) for ruptured arteriovenous malformations (AVMs) with and without endovascular embolization. No statistical difference was noted between the 2 groups (Log-rank test, *p* = 0.43). (**B**) Kaplan-Meier curves for repeat hemorrhage after GKRS for ruptured AVM with and without endovascular embolization. No statistical difference was noted between the 2 groups (Log-rank test, *p* = 0.21). (**C**) Kaplan-Meier curves for delayed cyst formation after GKRS for ruptured AVM with and without endovascular embolization. No statistical difference was noted between the 2 groups (Log-rank test, *p* = 0.83). (**D**) Kaplan-Meier curves for total obliteration confirmed by MRI/MRA or cerebral angiogram after GKRS for ruptured AVM belonging to SM grade III and IV with and without endovascular embolization. Statistical significance was noted between the 2 groups (Log-rank test, *p* = 0.0372).

In the embolized group, MRI/MRA or angiographic obliteration after GKRS was achieved in eight patients (47.1%). In the non-embolized group, MRI/MRA or angiographic obliteration after GKRS was achieved in 51 patients (63.0%). The difference was not significant according to the Chi-square test *(p =* 0.2232). The Kaplan-Meier curves for obliteration are shown in Figure 1A. The cumulative incidence of obliteration was not significantly different between the two groups (*p =* 0.4281, Log rank test). The Cox regression model for factors regarding time to obliteration is shown in Table 2. In univariate analyses for the two groups, those with a higher nidus volume VRAS, Pollock-Flickinger score, and SM grade before GKRS showed a significant inverse correlation for obliteration at the last follow-up.

### 3.3. Complications – Repeat Hemorrhage/Delayed Cyst Formation

Post-GKRS complications included repeat hemorrhage and cyst formation. In the embolized group, none of the 17 patients experienced repeat hemorrhage at the last follow-up. In the non-embolized group, eight repeat hemorrhage episodes were noted after GKRS. The difference was not significant according to the Chi-square test (*p =* 0.3439). The Kaplan-Meier curves for repeat hemorrhage are shown in Figure 1B. The cumulative incidence of hemorrhage was not significantly different between the two groups (*p =* 0.2099, Log rank test). The Cox regression model for factors regarding time to repeat hemorrhage is shown in Table 2. In the univariate analysis, those with a higher nidus volume before GKRS, remnant nidus volume after GKRS, higher VRAS, Pollock-Flickinger score, and SM grade showed a significantly higher likelihood of repeat hemorrhage at the last follow-up. Those with higher marginal dose and delta nidus volume (%) (nidus volume before GKRS–remnant nidus volume after GKRS) showed a significant inverse correlation for repeat hemorrhage at the last follow-up. 

In the embolized group, there was one patient with radiation-induced delayed cyst formation. In the non-embolized group, six patients showed delayed cyst formations after GKRS. The difference did not achieve statistical significance in the Chi-square test (*p =* 0.9999). The Kaplan-Meier curves for delayed cyst formation are shown in Figure 1C. The cumulative incidence of cyst formation was not significantly different between the two groups (*p =* 0.8341, Log rank test). The Cox regression model for factors regarding time to cyst is shown in Table 2. In the univariate analysis, those with a higher nidus volume before GKRS showed significance for delayed cyst formation at the last follow-up. Those with higher delta nidus volume showed a significant inverse correlation for delayed cyst formation at the last follow-up. 

### 3.4. Subgroup Analysis—SM Grade III&IV

Subgroup analysis was further performed on AVMs with SM grade III and IV. The non-embolized group consisted of 11 patients and the embolized group consisted of four patients. No statistically significant differences were detected between the two groups with respect to patient demographics, AVM characteristics, or radiosurgical parameters. No statistically significant difference was detected between the two groups with respect to obliteration, repeat hemorrhage, or delayed cyst formation *(p =* 0.5165, *p =* 0.5165, and *p =* 0.999, respectively). The cumulative incidence of repeat hemorrhage and delayed cyst formation were not significantly different between the two groups (*p =* 0.2374, *p =* 0.5637, respectively, Log rank test). However, statistical significance was observed in the cumulative incidence of obliteration (*p =* 0.0372, Log rank test) (Figure 1D). 

## 4. Case Illustration

### 4.1. Case 1 

**Figure 2:**
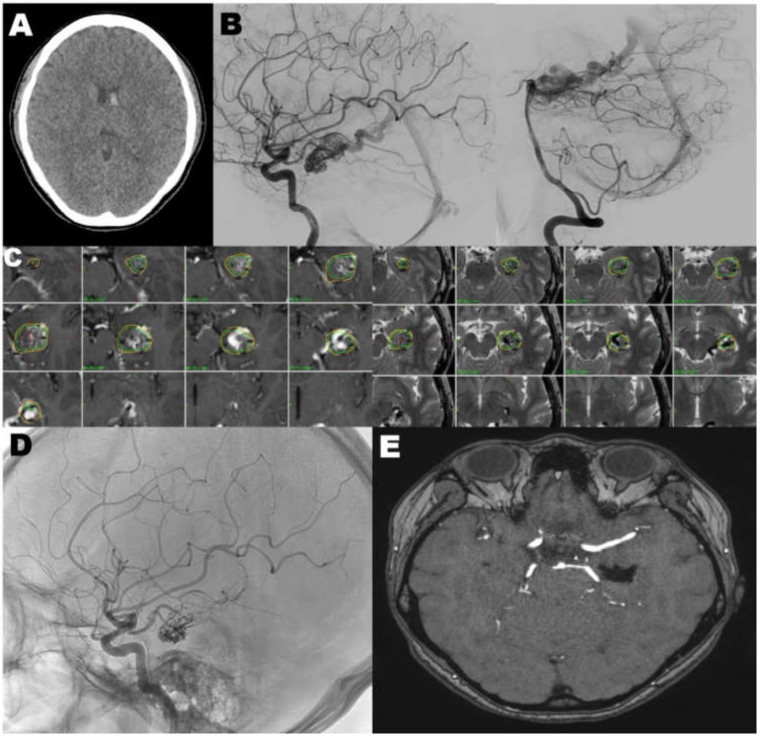
(**A**) The non-contrast enhanced computed tomography showed an acute intraventricular hemorrhage. (B) The digital subtraction angiography confirmed a Spetzler-Martin grade III AVM in the left temporal lobe with feeders from the left anterior choroidal artery and left posterior cerebral artery with venous ectasia, draining to the left basal vein of Rosenthal. (**C**) Gamma knife radiosurgery (3.30 cm^3^, 18 Gy to 50% of the isodense line). (**D**) The final angiogram after Onxy embolization showed near complete occlusion of the AVM nidus with multiple intranidal aneurysms. (**E**) The last follow up magnetic resonance imaging (24 months after GKRS) revealed neither residual nidus nor flow void.

A 28-year old patient was admitted to the emergency department with sudden onset of headache and nausea. The non-contrast enhanced CT scan revealed an acute intraventricular hemorrhage in the left lateral ventricle (Figure 2A). A subsequent CT angiography (CTA) suggested the presence of an AVM, which was confirmed by DSA, revealing a SM grade III AVM in the left temporal lobe with feeders from the left anterior choroidal artery and left posterior cerebral artery with venous ectasia, draining to the left basal vein of Rosenthal (Figure 2B). Also, multiple focal bulging lesions were found at the distal anterior choroidal artery, suggesting intranidal aneurysms. Twenty days after her first admission, the patient underwent GKRS (3.30cm^3^, 18 Gy to 50% of the isodense line; Figure 2C). Two days after GKRS, she underwent Onxy embolization (Figure 2D). The final angiogram showed near complete occlusion of the AVM nidus with multiple intranidal aneurysms. At her imaging follow-up examination (24 months after GKRS), the MRI/MRA revealed no flow void (Figure 2E).

### 4.2. Case 2

**Figure 3:**
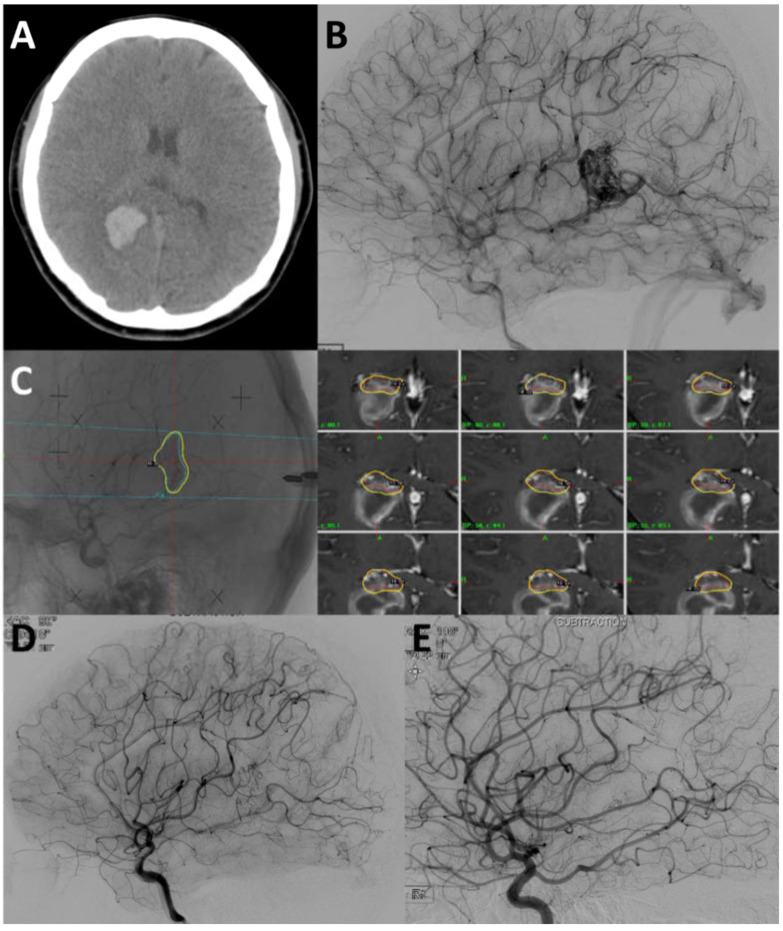
(**A**) The non-contrast enhanced computed tomography revealed an acute intracerebral hemorrhage in the right parieto-occipital lobe. (**B**) The digital subtraction angiography revealed a SM grade II AVM with feeders from the right parieto-occipital and calcarine arteries and draining to the vein of Galen. **(C)** Gamma knife radiosurgery (2.20 cm^3^, 16 Gy to 50% of the isodense line). (**D**) The final angiogram after NBCA 25% embolization showed near total occlusion of the AVM nidus. (**E**) The digital subtraction angiography (DSA) revealed complete obliteration of the AVM.

A 20-year old patient had presented with sudden onset of left homonymous hemianopsia. The non-contrast enhanced CT scan revealed an acute intracerebral hemorrhage in the right parieto-occipital lobe (Figure 3A). The DSA revealed a SM grade II AVM with feeders from the right parieto-occipital and calcarine arteries and draining to the vein of Galen (Figure 3B). Twelve days later, the patient underwent GKRS (2.20 cm^3^, 16 Gy to 50% of the isodense line; Figure 3C). Five days after GKRS, she underwent NBCA 25% embolization (Figure 3D). The final angiogram showed near total occlusion of the AVM nidus. The visual field defect had been recovered without any neurologic deficit. At her imaging follow-up examination (28 months after GKRS), the DSA revealed complete obliteration of the AVM (Figure 3E). 

## 5. Discussion

We started this study to compare the outcomes of ruptured AVMs at a higher risk of subsequent hemorrhage treated with and without post-GKRS embolization and to investigate the potential benefits of post-GKRS embolization. We hypothesized that the potential benefits of post-GKRS embolization would lower the risk of subsequent hemorrhage and increase the rate of obliteration of previously ruptured AVMs.

Univariate analysis showed that patients with a larger nidus volume, higher SM grade, higher VRAS, and higher Pollock-Flickinger score AVMs have a significantly lower chance of obliterating a nidus (*p* = 0.003, 0.004, <0.001, and <0.001, respectively) (Table 2). AVM volume reportedly has an inverse relationship with obliteration after GKRS [10,21,22,23,24,25]. The median nidus volume was larger in the embolized group compared to the non-embolized group, although this was not statistically significant (Table 1). The previous study reported that a lower SM grade, lower BRAS, and lower Pollock-Flickinger score were statistically significant for predicting the probability of AVM obliteration [23]. In the present study, there were statistically significant differences between the embolized and non-embolized group with respect to the SM grade distribution and Pollock-Flickinger score. Thus, post-GKRS embolization could offer a high obliteration of AVM with larger nidus volume and higher grading scores, which might have a lower chance of obliteration without post-GKRS embolization.

Using univariate analysis, the marginal dose, nidus volume, SM grade, VRAS, and Pollock-Flickinger score were identified as predictive factors for subsequent hemorrhage in the present study (Table 2). Our findings are consistent with Huo et al. [23]. The authors suggested that deep location, aneurysm, increased volume, decreased maximum dose, decreased marginal dose, higher VRAS score, and higher Pollock-Flickinger score were associated with postradiosurgical hemorrhage. In our study, there was no significant difference between the two groups regarding VRAS. However, the embolized group included more AVMs with higher SM grade and Pollock-Flickinger score, which are those with a higher probability of repeat hemorrhage. Although the univariate analysis in our study did not identify the presence of aneurysm as a predictor of latent period hemorrhage after GKRS, the embolized group included more AVMs with intranidal or flow-related aneurysm. The risk of intracranial hemorrhage in patients with AVMs and associated aneurysms is higher than that in patients with AVMs alone [26,27]. Furthermore, patients with aneurysms associated with ruptured AVMs are at higher risk for subsequent hemorrhage. Thus, we recommend aggressive management to treat aneurysms associated with AVMs by endovascular embolization. This treatment rationale is based on reports of aneurysms disappearing after the successful obliteration of AVMs [26,28]. In addition, embolization can reduce the flow in shunting vessels by blocking high-flow fistulas [29]. The use of embolization with permanent agents can play an important role in high-grade AVMs with intranidal or perinidal aneurysms or fistulae [30]. Furthermore, the selective embolization of high-flow arterial feeders can alleviate flow-related neurological symptoms or deficits. Although the post-GKRS hemorrhage rate was not significantly different between the two groups, none of the 17 patients in the embolized group experienced a latent period repeat hemorrhage after GKRS. Previous studies have suggested a dose-volume-tolerance relationship for the occurrence of complications [15,31]. We also found that radiation dose and nidus volume were critical predictors for the occurrence of repeat hemorrhage. Nidus size may be reduced with endovascular embolization to reduce the complication rate and achieve complete obliteration of ruptured AVMs.

Several studies have analyzed the effect of prior embolization on AVM radiosurgery outcomes; they proposed that embolization prior to GKRS may reduce the final obliteration rate. Kano et al. demonstrated that prior embolization reduced the total obliteration rate after GKRS [10]. Pollock et al. investigated factors related to successful outcomes after radiosurgery in 220 patients with AVM, including 40 who underwent initial embolization. Embolization prior to GKRS was identified as a negative predictor for complete nidus obliteration [32]. Several potential mechanisms have been suggested for the diminished rate of GKRS-induced obliteration with prior embolization, as mentioned in the Introduction section. The unfavorable effects of pre-GKRS embolization on obliteration are also attributed to nidus angioarchitectural complexity. Larger AVMs with more complex angioarchitecture are usually selected for combined management, consistent with the distribution of AVMs identified in the two groups of our study, regarding nidus volume, SM grade, and Pollock-Flickinger score. Nidus angioarchitecture may be a significant factor in the statistical evaluation of the effect of prior embolization on AVM obliteration after GKRS because of the inverse correlation between nidus angioarchitectural complexity and post-GKRS obliteration rate [21]. Thus, a higher angioarchitectural complexity may partially explain the lower obliteration rates observed in embolized AVMs [21]. High-grade AVMS, those of SM grade III or higher, are extremely difficult to treat by single-session GKRS alone [33]. Despite the small sample size (11 patients in the non-embolized group and four patients in the embolized group), the subgroup analysis of SM grades III and IV showed a significant difference in the cumulative incidence of obliteration (*p =* 0.0372, Log rank test) (Figure 1D).

In the present study, it was unusual to demonstrate six (7.4%) delayed cyst formation in the non-embolized group after GKRS with the short latency period. In the systematic review by Ilyas et al., the overall rate of post-SRS cyst formation was 3.0%, which is half the percentage in our report, and the mean latency period to post-SRS cyst formation was 78 months (6.5 years) [34]. However, several studies have showed cyst formation after GKRS with a short latency period [10,35,36,37,38,39,40]. Hasegawa et al. presented that prior hemorrhage before GKRS was a significant factor for late AREs including cyst formation [41]. Pan et al. also found statistical significance between prior AVM hemorrhage and cyst formation [42]. The cause of the development of cyst formation remains unclear. Histopathological examination of resected cysts suggests that cyst formation occurs secondary to a chronic, organized response to radiation-induced vascular telangiectasia and wall damage, which promotes microhemorrhage and protein exudation [36]. Among the possible mechanisms suggested by Maiuri et al., a hypothesis describing a cyst formed by intracerebral hemorrhage itself is one possible mechanism for cyst formation. It is possible that prior AVM hemorrhage increases tissue susceptibility to damage, thereby increasing the risk of cyst formation. The present study only concerns ruptured AVMs. One possible reason for the high incidence (7.4%) of cyst formation with a relatively short latency period is that we only included patients who had previously undergone hemorrhage before GKRS. 

### Study Limitations 

The major limitation of the present study includes the retrospective review at a single institution with a small number of patients (17 patients with embolization). Regarding the subgroup analysis performed on AVMs with SM grade III and IV, this consisted of 11 patients in the non-embolized group and 4 patients in the embolized group. Although statistical significance was observed in cumulative incidence of obliteration between the two groups, it may not be considered to prove the clinical significance because of the small sample size. The selection bias may play a critical role in patient selection and the decision to choose treatment because larger AVMs with more complex angioarchitecture are usually selected for combined management. In addition, the treatment bias associated with endovascular embolization being decided by “accessibility” was unavoidable. Lastly, our institution started to treat larger AVMs with staged-GKRS rather than single-session GKRS. In this study, only first single-session GKRS data of patients were used for analysis. 

## 6. Conclusions

In the present study, predictive factors for the obliteration of ruptured AVMs were nidus volume, SM grade, VRAS, and Pollock-Flickinger score. In addition, our study showed that marginal dose, nidus volume, SM grade, VRAS, and Pollock-Flickinger score significantly affected subsequent hemorrhage after GKRS. Although the current study demonstrated similar results in patients who underwent GKRS with and without embolization, the embolized group included more AVMs with larger nidus volume, higher SM grade, Pollock-Flickinger score, and aneurysm, which have a lower chance of obliteration and a higher probability of repeat hemorrhage. GKRS followed by embolization appears to be a beneficial approach for the treatment of ruptured AVMs that are at risk for obliteration failure and repeat hemorrhage during the latency period after single-session GKRS alone. Further studies involving a larger number of cases and continuous follow-up are necessary to confirm our conclusions.

## Figures and Tables

**Table 1:** Comparison of baseline demographic data, arteriovenous malformation (AVM) characteristics, and radiosurgical parameters.

	Non-Embolized (*n* = 81)	Embolized (*n* = 17)	*p*-Value
Median age	22 (14, 38)	39 (29, 48)	**0.0045**
Sex			0.3073
Male	49 (60.5%)	8 (47.1%)	
Female	32 (39.5%)	9 (52.9%)	
Median FU time, month	47 (36, 67)	47 (41, 59)	0.9738
Median marginal dose, Gy	16 (15, 18)	16 (16, 17)	0.6685
Median isodense line	50% (50.0%)	50% (50.0%)	
Median nidus volume, cm^3^	2.14 (0.87, 5.41)	3.30 (2.10, 5.70)	0.4084
Median remnant nidus volume, cm^3^	0 (0, 0.40)	0.29 (0, 0.90)	0.1792
Median delta nidus volume, %	100 (89.2, 100)	89.2 (81.8, 100)	0.1221
VRAS			0.1161
0	0 (0%)	0 (0%)	
1	34 (42.0%)	3 (17.6%)	
2	13 (16.0%)	3 (17.6%)	
3	20 (24.7%)	8 (47.2%)	
4	14 (17.3%)	3 (17.6%)	
Pollock-Flickinger score	0.95 (0.59, 1.37)	1.30 (1.05, 1.95)	**0.0316**
<1	44 (54.3%)	4 (23.5%)	
1.01–1.50	20 (24.7%)	5 (29.4%)	
1.51–2.00	6 (7.4%)	5 (29.4%)	
>2	11 (13.6%)	3 (17.7%)	
SM grade			**0.0023**
I	42 (51.8%)	2 (11.8%)	
II	28 (34.6%)	11 (64.7%)	
III	11 (13.6%)	3 (17.6%)	
IV	0 (0%)	1 (5.9%)	
Eloquence			**0.029**
Noneloquent	67 (82.7%)	10 (58.8%)	
Eloquent	14 (17.3%)	7 (41.2%)	
Venous drainage			
Superficial only	62 (76.5%)	10 (58.8%)	0.132
Deep	19 (23.5%)	7 (41.2%)	
Presence of Aneurysm	12 (14.8%)	8 (47.0%)	**0.0060**
Intranidal	12 (14.8%)	6 (35.3%)	
Flow-related	0 (0%)	2 (11.7%)	

FU = follow up, SM = Spetzler-Martin, VRAS = Virginia Radiosurgery AVM scale. Delta nidus volume = (nidus volume – remnant nidus volume/nidus volume) x100, Descriptive statistics are presented as medians (Q1: cumulative percentage of 25%, Q3: cumulative percentage of 75%); Boldface type indicates statistical significance (*p* < 0.05).

**Table 2:** Predictive factors for time to obliteration, repeat hemorrhage, and delayed cyst formation of arteriovenous malformations (AVMs) in 98 patients: Cox regression.

	Obliteration			Hemorrhage			Cyst Formation		
	Univariate Analysis			Univariate Analysis			Univariate Analysis		
Factors	HR	CI	*p*-Value	HR	CI	*p*-Value	HR	CI	*p*-Value
Post GKRS Embo	0.746	(0.354, 1.574)	0.4418	0.302	(0.015, 6.220)	0.4377	0.798	(0.096, 6.633)	0.8346
Age	0.992	(0.977, 1.008)	0.3486	1.010	(0.971, 1.049)	0.6305	0.990	(0.942, 1.040)	0.6785
Female sex	1.268	(0.757, 2.122)	0.3671	0.462	(0.093, 2.291)	0.3448	1.097	(0.245, 4.921)	0.9033
Marginal dose, Gy	1.010	(0.983, 1.039)	0.4621	0.438	(0.259, 0.741)	**0.0021**	0.605	(0.340, 1.075)	0.0865
Nidus volume, cm^3^	0.981	(0.971, 0.991)	**0.0003**	1.010	(1.006, 1.015)	**<0.0001**	1.009	(1.002, 1.015)	**0.0119**
Remnant nidus volume, cm^3^	0.940	(0.737, 1.199)	0.6187	1.013	(1.006, 1.021)	**0.0003**	1.013	(0.998, 1.027)	0.0855
Delta nidus volume, %	20.168	(0.132, 3085.252)	0.2418	0.956	(0.933, 0.979)	**0.0002**	0.959	(0.924, 0.996)	**0.0304**
Higher SM grade	0.480	(0.319, 0.721)	**0.0004**	3.082	(1.359, 6.986)	**0.0070**	1.216	(0.438, 3.380)	0.7073
Higher VRAS	0.579	(0.449, 0.747)	**<0.0001**	3.759	(1.448, 9.757)	**0.0065**	1.192	(0.597, 2.378)	0.6185
Higher Pollock–Flickinger score	0.390	(0.246, 0.619)	**<0.0001**	2.819	(1.674, 4.746)	**<0.0001**	2.066	(0.992, 4.304)	0.0525
Aneurysm	1.031	(0.546, 1.947)	0.9255	1.276	(0.257, 6.321)	0.7655	0.670	(0.080, 5.595)	0.7113

HR: Hazard ratio, CI: confidence interval, VRAS = Virginia Radiosurgery AVM scale GKRS = Gamma knife radiosurgery, Embo = embolization, SM = Spetzler-Martin; Delta nidus volume = (nidus volume – remnant nidus volume / nidus volume) × 100; Descriptive statistics are presented as medians (Q1: cumulative percentage of 25%, Q3: cumulative percentage of 75%); Boldface type indicates statistical significance ( *p* < 0.05).

## References

[1] Kano H., Kondziolka D., Flickinger J.C., Yang H.C., Flannery T.J., Awan N.R., Niranjan A., Novotny J., Lunsford L.D. (2012). Stereotactic radiosurgery for arteriovenous malformations, part 3: Outcome predictors and risks after repeat radiosurgery. J. Neurosurg..

[2] Auger R.G., Wiebers D.O. (1992). Management of unruptured intracranial arteriovenous malformations: A decision analysis. Neurosurgery.

[3] Brown R.D., Wiebers D.O., Forbes G., O’Fallon W.M., Piepgras D.G., Marsh W.R., Maciunas R.J. (1988). The natural history of unruptured intracranial arteriovenous malformations. J. Neurosurg..

[4] Pollock B.E., Flickinger J.C., Lunsford L.D., Bissonette D.J., Kondziolka D. (1996). Factors that predict the bleeding risk of cerebral arteriovenous malformations. Stroke.

[5] Pollock B.E., Flickinger J.C., Lunsford L.D., Bissonette D.J., Kondziolka D. (1996). Hemorrhage risk after stereotactic radiosurgery of cerebral arteriovenous malformations. Neurosurgery.

[6] Izawa M., Chernov M., Hayashi M., Iseki H., Hori T., Takakura K. (2009). Combined management of intracranial arteriovenous malformations with embolization and gamma knife radiosurgery: Comparative evaluation of the long-term results. Surg. Neurol..

[7] Solomon R.A., Connolly E.S. (2017). Arteriovenous malformations of the brain. N. Engl. J. Med..

[8] Da Costa L., Wallace M.C., Ter Brugge K.G., O’Kelly C., Willinsky R.A., Tymianski M. (2009). The natural history and predictive features of hemorrhage from brain arteriovenous malformations. Stroke.

[9] Todnem N., Ward A., Nahhas M., Vender J.R., Alleyne C.H., Rahimi S.Y. (2019). A retrospective cohort analysis of hemorrhagic arteriovenous malformations treated with combined endovascular embolization and gamma knife stereotactic radiosurgery. World Neurosurg..

[10] Kano H., Kondziolka D., Flickinger J.C., Park K.J., Iyer A., Yang H.C., Liu X., Monaco E.A., Niranjan A., Lunsford L.D. (2012). Stereotactic radiosurgery for arteriovenous malformations after embolization: A case-control study. J. Neurosurg..

[11] Friedman W.A., Bova F.J. (1992). Linear accelerator radiosurgery for arteriovenous malformations. J. Neurosurg..

[12] Yamamoto M., Jimbo M., Hara M., Saito I., Mori K. (1996). Gamma knife radiosurgery for arteriovenous malformations: Long-term follow-up results focusing on complications occurring more than 5 years after irradiation. Neurosurgery.

[13] Van Beijnum J., van der Worp H.B., Buis D.R., Al-Shahi Salman R., Kappelle L.J., Rinkel G.J., van der Sprenkel J.W., Vandertop W.P., Algra A., Klijn C.J. (2011). Treatment of brain arteriovenous malformations: A systematic review and meta-analysis. JAMA.

[14] Huo X., Jiang Y., Lv X., Yang H., Zhao Y., Li Y. (2015). Targeted embolization reduces hemorrhage complications in partially embolized cerebral avm combined with gamma knife surgery. Interv. Neuroradiol..

[15] Ogilvy C.S. (1990). Radiation therapy for arteriovenous malformations: A review. Neurosurgery.

[16] Kwon Y., Jeon S.R., Kim J.H., Lee J.K., Ra D.S., Lee D.J., Kwun B.D. (2000). Analysis of the causes of treatment failure in gamma knife radiosurgery for intracranial arteriovenous malformations. J. Neurosurg..

[17] Andrade-Souza Y.M., Ramani M., Scora D., Tsao M.N., terBrugge K., Schwartz M.L. (2007). Embolization before radiosurgery reduces the obliteration rate of arteriovenous malformations. Neurosurgery.

[18] Sure U., Battenberg E., Dempfle A., Tirakotai W., Bien S., Bertalanffy H. (2004). Hypoxia-inducible factor and vascular endothelial growth factor are expressed more frequently in embolized than in nonembolized cerebral arteriovenous malformations. Neurosurgery.

[19] Hung Y.C., Mohammed N., Eluvathingal Muttikkal T.J., Kearns K.N., Li C.E., Narayan A., Schlesinger D., Xu Z., Sheehan J.P. (2019). The impact of preradiosurgery embolization on intracranial arteriovenous malformations: A matched cohort analysis based on de novo lesion volume. J. Neurosurg..

[20] Lee C.C., Chen C.J., Ball B., Schlesinger D., Xu Z., Yen C.P., Sheehan J. (2015). Stereotactic radiosurgery for arteriovenous malformations after onyx embolization: A case-control study. J. Neurosurg..

[21] Oermann E.K., Ding D., Yen C.P., Starke R.M., Bederson J.B., Kondziolka D., Sheehan J.P. (2015). Effect of prior embolization on cerebral arteriovenous malformation radiosurgery outcomes: A case-control study. Neurosurgery.

[22] Ding D., Yen C.P., Starke R.M., Xu Z., Sun X., Sheehan J.P. (2014). Outcomes following single-session radiosurgery for high-grade intracranial arteriovenous malformations. Br. J. Neurosurg..

[23] Huo X., Jiang Y., Lv X., Yang H., Zhao Y., Li Y. (2016). Gamma knife surgical treatment for partially embolized cerebral arteriovenous malformations. J. Neurosurg..

[24] Starke R.M., Kano H., Ding D., Lee J.Y., Mathieu D., Whitesell J., Pierce J.T., Huang P.P., Kondziolka D., Yen C.P. (2017). Stereotactic radiosurgery for cerebral arteriovenous malformations: Evaluation of long-term outcomes in a multicenter cohort. J. Neurosurg..

[25] Lunsford L.D., Kondziolka D., Flickinger J.C., Bissonette D.J., Jungreis C.A., Maitz A.H., Horton J.A., Coffey R.J. (1991). Stereotactic radiosurgery for arteriovenous malformations of the brain. J. Neurosurg..

[26] Thompson R.C., Steinberg G.K., Levy R.P., Marks M.P. (1998). The management of patients with arteriovenous malformations and associated intracranial aneurysms. Neurosurgery.

[27] Wilkins R.H. (1985). Natural history of intracranial vascular malformations: A review. Neurosurgery.

[28] Batjer H., Suss R.A., Samson D. (1986). Intracranial arteriovenous malformations associated with aneurysms. Neurosurgery.

[29] Hodgson T.J., Kemeny A.A., Gholkar A., Deasy N. (2009). Embolization of residual fistula following stereotactic radiosurgery in cerebral arteriovenous malformations. Ajnr. Am. J. Neuroradiol..

[30] Ogilvy C.S., Stieg P.E., Awad I., Brown R.D., Kondziolka D., Rosenwasser R., Young W.L., Hademenos G. (2001). Aha scientific statement: Recommendations for the management of intracranial arteriovenous malformations: A statement for healthcare professionals from a special writing group of the stroke council, american stroke association. Stroke.

[31] Friedman W.A., Bova F.J., Mendenhall W.M. (1995). Linear accelerator radiosurgery for arteriovenous malformations: The relationship of size to outcome. J. Neurosurg..

[32] Pollock B.E., Flickinger J.C., Lunsford L.D., Maitz A., Kondziolka D. (1998). Factors associated with successful arteriovenous malformation radiosurgery. Neurosurgery.

[33] Spetzler R.F., Martin N.A. (1986). A proposed grading system for arteriovenous malformations. J. Neurosurg..

[34] Ilyas A., Chen C.J., Ding D., Mastorakos P., Taylor D.G., Pomeraniec I.J., Lee C.C., Sheehan J. (2018). Cyst formation after stereotactic radiosurgery for brain arteriovenous malformations: A systematic review. J. Neurosurg..

[35] Bir S.C., Ambekar S., Maiti T.K., Nanda A. (2015). Clinical outcome and complications of gamma knife radiosurgery for intracranial arteriovenous malformations. J. Clin. Neurosci..

[36] Shuto T., Yagishita S., Matsunaga S. (2015). Pathological characteristics of cyst formation following gamma knife surgery for arteriovenous malformation. Acta Neurochir..

[37] Bowden G., Kano H., Tonetti D., Niranjan A., Flickinger J., Arai Y., Lunsford L.D. (2014). Stereotactic radiosurgery for sylvian fissure arteriovenous malformations with emphasis on hemorrhage risks and seizure outcomes. J. Neurosurg..

[38] Matsuo T., Kamada K., Izumo T., Hayashi N., Nagata I. (2014). Cyst formation after linac-based radiosurgery for arteriovenous malformation: Examination of predictive factors using magnetic resonance imaging. Clin. Neurol. Neurosurg..

[39] Kano H., Kondziolka D., Flickinger J.C., Yang H.C., Flannery T.J., Niranjan A., Novotny J., Lunsford L.D. (2012). Stereotactic radiosurgery for arteriovenous malformations, part 5: Management of brainstem arteriovenous malformations. J. Neurosurg..

[40] Kim M.S., Lee S.I., Sim J.H. (1999). A case of very large cyst formation with gamma knife radiosurgery for an arteriovenous malformation. Stereotact. Funct. Neurosurg..

[41] Hasegawa T., Kato T., Naito T., Tanei T., Torii J., Ishii K., Tsukamoto E., Hatanaka K.C., Sugiyama T. (2019). Long-term outcomes for pediatric patients with brain arteriovenous malformations treated with gamma knife radiosurgery, part 2: The incidence of cyst formation, encapsulated hematoma, and radiation-induced tumor. World Neurosurg..

[42] Pan H.C., Sheehan J., Stroila M., Steiner M., Steiner L. (2005). Late cyst formation following gamma knife surgery of arteriovenous malformations. J. Neurosurg..

